# The Association between Job Quality Profiles and Work-Life Balance among Female Employees in Korea: A Latent Profile Analysis

**DOI:** 10.3390/ijerph18041672

**Published:** 2021-02-09

**Authors:** Eun Young Choi, Seung-Hye Choi, Haeyoung Lee

**Affiliations:** 1 Department of Nursing, Graduate School of Chung-Ang University, Seoul 06974, Korea; 11351@naver.com; 2 College of Nursing, Gachon University, Incheon 21936, Korea; 3 Red Cross College of Nursing, Chung-Ang University, Seoul 06974, Korea

**Keywords:** job quality, work-life balance, employee, latent profile analysis

## Abstract

Women’s participation in society has been increasing; however, they often remain overloaded with housework, and this gender role difference can hinder their work-life balance in Korea. Therefore, this study classified latent profiles according to job quality indices for South Korean female employees and examined the characteristics of each profile and how they affect work-life balance. This study was a secondary analysis of data collected through the fifth Korean Working Conditions Survey in South Korea. The Bayesian information criterion, entropy, and the Lo-Mendell-Rubin adjusted likelihood ratio test were used to determine the number of latent profiles. Chi-square tests were conducted to understand the characteristics of each profile. Comparisons between work-life balance and the latent profiles were made using the Bolck-Croon-Hagenaars method. Female employees in South Korea were classified into five profiles: “high-flying”, “smooth”, ”footloose”, ”strict” and “manual”. The “footloose” profile showed the most positive work-life balance, and the “manual” profile had the highest level of work-family conflict. Therefore, policies and social supports should be created with the aim of improving the implementation of current strategies promoting work-life balance to better fit each working condition.

## 1. Introduction

Job quality is important to individuals and is a central policy concern as countries strive to promote economic growth and manage the threat of a low birth rate and an aging population [1]. It is important to maintain and improve job quality to make work sustainable and enable people to work longer. According to South Korean employment-related indicators in 2019, temporary employment accounted for 24% of all workers; this was the fourth highest rate of countries in the Organization for Economic Co-operation and Development (OECD) [2], and the rate of low-pay workers was 17%, the sixth highest in the OECD [3]. Although minimum wage relative to average wages of full-time workers was the sixth highest in the OECD [4], the ratio of employees working below the minimum wage was 16.5% [5]. In addition, the average annual hours actually worked per worker was 1967, the third highest in the OECD and 300 h longer than the OECD average [6]. The proportion of employees who were satisfied with their overall job in their workplace was low, at 32.3% [7].

These phenomena also differ greatly by gender. Working conditions for women were found to be poorer compared to those for men because women are marginalized or have unstable positions in the labor market due to the stereotype in the patriarchal system that men must be responsible for the family and to the gender segmentation of the labor market. Conditions for women workers, such as having a child, were found to be poor [8,9]. In 2018, the gender employment gap was nearly 18%, the fourth largest in the OECD, and the gender wage gap was about 34%, which is the widest in the OECD against an OECD average of about 13% [6]. Even though gender equality is not only a basic human right but also an essential foundation for a peaceful, prosperous, and sustainable world [10], it is not guaranteed in the Korean labor market.

Research over the past several years has emphasized the importance of work-life balance, and it has been reported that a lack of such balance can negatively affect the quality of life and mental health of individuals and their families [11]. From the viewpoint that the quality of work and the quality of family life should be managed from one integrated perspective rather than as separate dimensions, even in organizations, work–life balance has been established as a very important concept [12,13]. However, despite institutional efforts such as companies’ family-friendly systems [14] and the government’s work-family compatibility support policy [15], work-life balance has not been successfully realized in South Korea. According to the results of a 2020 survey by the Better Life Index, a comprehensive quality of life measuring instrument of the OECD, South Korea’s work-life balance is very low, ranking 37th out of a total of 40 countries [16].

In particular, in the case of female workers, quality of work shows a close association with work-life balance [17,18]. Women’s participation in society has been increasing; however, they often remain overloaded with housework [19], and this gender role difference can hinder their work-life balance in Korea [20]. In South Korea, on average, employed women” spent 2 h and 24 min on housework in 2019, which was 2 h and 13 min longer than that of men in dual-income households. In 2019, the hourly wage of female wage workers was 69.4% of that of men, and while the gender gap is narrowing, the wages of female workers are still lower compared to those of male workers [21]. Excessive working hours, irregular working time, and work-related time pressure can increase the burden of raising children [22], and job stressors such as a high job burden and low job autonomy may lead to work-life imbalance [23]. In particular, for female employees, research findings have indicated that the higher the physical demand, the more work-life conflict experienced [24].

The job demand-resources (JD-R) model is a useful theoretical approach for understanding the relationship between work quality and work-life balance [25,26,27]. This model divides the characteristics of work into demands and resources [28]. Job demands refer to the features of the job that demand physical and mental effort. Job demands are not necessarily negative, but if excessive effort and expense are required to meet those demands, they can become a stress factor and cause negative reactions. Job demands include time pressure, excessive workloads, role ambiguity, and conflicts in interpersonal relationships experienced when carrying out the job [29,30]. Job resources refer to the characteristics of a job that reduce the costs associated with job demands or motivate workers in achieving goals. Job resources include factors such as job autonomy, participation in decision making, diversity of skills, and feedback provision as well as factors at the organizational level such as support from supervisors and colleagues, wage levels, job stability, and organizational fairness [30,31,32]. According to this model, the level of work-life balance of workers becomes high when job demands are low, and when there are many job resources available, even if job demands are high.

In the JD-R model, job demand and job resources are related to each other, but the relationship varies according to the job or job characteristics [33]. Therefore, many studies using this model organize the dimensions of job demands and job resources according to the population before carrying out an analysis [34,35,36]. In this study, job quality indices were composed of seven areas based on the EWCS report [37]. These indices comprehensively reflect job demands and resources, or the processes that affect them, in various aspects of job characteristics, but according to the JD-R model, we classified the physical environment, work intensity, working time quality, skills, and discretion into demand factors and social environment, prospects, and earning into job resources factors.

Meanwhile, the quality of a job is multifaceted and requires consideration of both the objective situations and the subjective evaluation of the workers who judge the situations [37]. Previous studies that mainly approached working conditions as individual or integrated variables did not sufficiently reflect the multidimensional interrelationship between work conditions and work-life balance [38,39,40]. Latent profile analysis has an advantage in that it enables the classification of the potential types of respondents into groups while comprehensively considering the levels of various observation variables and analyzing the differential effects of the various patterns of responses regarding work quality on workers. In addition, it enables the identification of the distributions of sociodemographic characteristics such as age and career, which are closely related to the characteristics of work, by subgroup, as well as occupational characteristics, such as occupations and positions, alongside the differences in work–life balance. Consequently, tailored interventions that fit the characteristics of heterogeneous subgroups according to the quality of work will be possible.

**Figure 1:**
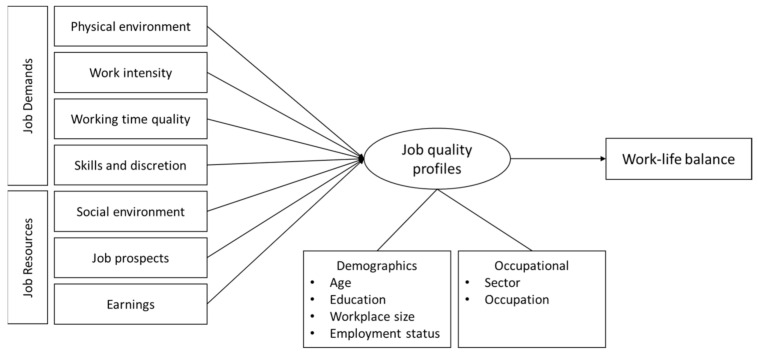
Theoretical framework of this study.

Therefore, this study attempted to categorize subgroups according to the seven types of work quality of female workers, investigate the sociodemographic and professional characteristics of each group, and identify the effects of these types on work–life balance (Figure 1). The research questions for this study are as follows: (1) How do the latent profiles appear according to the quality of work of female workers? (2) How are the sociodemographic and occupational characteristics distributed by job quality latent profile? (3) What are the differences in the levels of work–life balance according to job quality latent profile?

## 2. Materials and Methods

### 2.1. Participants

This study utilized data from the Korean Working Conditions Survey (KWCS) conducted by the Korea Occupational Safety and Health Agency. The KWCS collected data [41] with an aim to understand factors of overall working conditions, such as working types, employment status, occupational groups, sectors, exposure to risk factors, and job security, for employees of at least 15 years of age nationwide (e.g., self-employed workers, business owners, employees). The present study used data from the fifth KWCS (2017) and included 15,723 female employees out of a total of 50,205 survey participants.

### 2.2. Measures

#### 2.2.1. Job Quality Indices

Seven job quality indices were used to identify latent profiles: (1) physical environment, (2) work intensity, (3) working time quality, (4) social environment, (5) skills and discretion, (6) prospects, and (7) earnings.

“Physical environment” was measured with 15 items for ambient, biological, chemical, and ergonomic risks in participants” workplaces. “Work intensity” was measured with 12 items for quantitative and emotional demands and pace determinants of the job. “Working time quality” was measured with 19 items for duration, atypical working time, working time arrangements, and flexibility. “Social environment” was measured with 15 items for management quality, social support, and adverse social behaviors such as bullying and violence in the workplace. “Skills and discretion” was measured with 15 items for cognitive dimension, decision latitude, organizational participation, and training. “Prospects” was measured with seven items for employment status, career prospects, and job security. “Earnings” was measured using average monthly income received from work.

Each item of six of the job quality indices was measured on a dichotomous or 5–7-point Likert scale, and participants responded to one item, “earnings”, on a continuous scale.

#### 2.2.2. Demographic and Occupational Characteristics

The demographic and occupational characteristics that were selected were age, education, sector, occupation, workplace size, and employee status, and they were used to examine the characteristics of the classified latent profiles. In the 5th KWCS, sectoral analysis was conducted based on the Korean Standard Industrial Classification (KSIC) and occupational analysis on the Korean Standard Classification of Occupation (KSCO). In this study, 21 sections of the KSIC were reclassified into 10 categories based on the 6th EWCS.

#### 2.2.3. Work-Life Balance

Work-life balance was measured with one positive item and five negative items. An example of a positive item was whether a participant’s working hours were appropriate for having a successful family or social life. Responses were measured on a four-point Likert scale ranging from “not suitable at all” to “very suitable”, with higher scores indicating work-life balance. The five negative items consisted of three work-family conflict items and two family–work conflict items. The items about work-family conflict were: “I worry about work even when I am not working,” “I am too tired to do household chores after work,” and “I do not have enough time to spend with my family due to my work.” The items about family–work conflict were: “I do not have enough time to work because of what happens at home,” and “I feel that I am unable to devote time to work because of my responsibility to my family.” Responses were measured on a five-point Likert scale ranging from “not at all” to “always”; higher scores indicated more conflict.

### 2.3. Data Analysis

Descriptive analyses were conducted to understand means and standard deviations of all variables using SPSS (IBM, Armonk, NY, USA). Latent profile analysis (LPA) was used to determine latent profiles based on the seven job qualities using Mplus 8.4 (Muthén & Muthén, Los Angeles, CA, USA). Before the original LPA, missing data were imputed using mean values. The six job quality indices, excluding earnings, were calculated as mean index scores by converting the responses to each item into scores ranging from 0–100 points, with positive responses receiving higher scores. For earnings, responses were converted into logs and applied. In LPA, the scores of all job quality indices were standardized using DEFINE in Mplus. Z-score is a standardized score that subtracts the mean from each value and then dividing by the standard deviation [42].

We started with a two-profile solution and increased the number of extracted profiles until the model fit no longer improved [43]. To determine the number of latent profiles, we comprehensively considered statistical and theoretical criteria [43]. Bayesian information criterion (BIC) [44], entropy [45], and the Lo-Mendell-Rubin adjusted likelihood ratio test (LMR-LRT) were used as statistical criteria [46]. For BIC, lower values indicate better model fit. Entropy refers to the accuracy of individuals” assignments to different groups, and values higher than 0.70 and close to 1 are preferred. LMR-LRT compares k group models to k^−1^ group models and shows that lower significance values (*p* < 0.05) have a better fit. To comprehensively consider interpretability, the results of the sixth EWCS were used as a reference for theoretical criteria.

After determining the number of profiles, we conducted a chi-square test to understand the demographic and occupational characteristics of each profile using most-likely class membership, which was obtained based upon posterior distribution [47]. Finally, the associations between work-life balance and latent profiles were compared using the Bolck-Croon-Hagenaars (BCH) auxiliary method [48]. The BCH auxiliary method determines whether there are statistically significant differences between the profiles on an outcome variable of interest, again accounting for possible errors in classification [47].

### 2.4. Ethical Considerations

The study was approved by the Institutional Review Board (IRB no 1041078-201906-HRSB-185-01) of Chung-Ang University on 14 June 2019. Potential ethical issues were addressed, including plagiarism, misconduct, data fabrication and/or falsification, double publication and/or submission, and redundancy.

## 3. Results

### 3.1. Job Quality Profile Models

To classify profiles according to the seven job quality indices, latent profiles were examined in consideration of various statistical criteria (Table 1). As the number of latent profiles increased from two to six, the BIC gradually decreased, and all comparison results of the LMR-LRT model were found to be significant. However, the BIC showed a smaller decrease when the number of potential profiles changed from four to five, compared to when the number changed from five to six. When the participants were classified into six profiles, a group with a potential profile classification rate of 0.5% was identified; thus, it was difficult to regard the group classification as being representative. As a result of comprehensively considering the various criteria above, a model with five potential profiles was selected as the final one.

### 3.2. Five Job Quality Profiles

**Figure 2:**
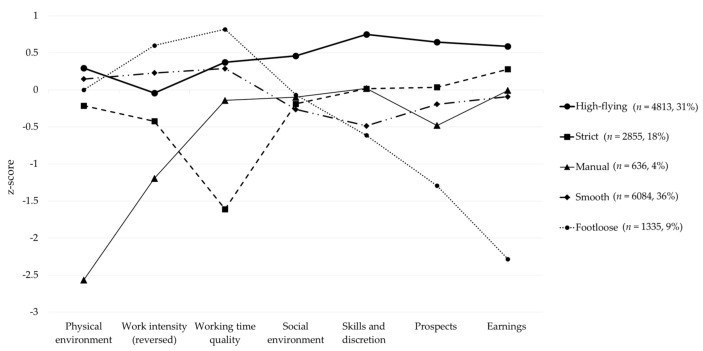
Five job quality profiles by job quality indices.

Regarding job quality patterns, female employees were classified into five profiles: “high-flying”, “smooth”, “footloose”, “strict” and “manual” (Figure 2).

The “high-flying” profile was a subgroup to which 31% (*n* = 4813) of the female employees belonged. These employees received the highest scores on most indicators. In particular, their incomes and job prospects were high, and they performed work that required highly skilled techniques. Furthermore, the intensity of their work was high, and the quality of their working time was low.

The “smooth” profile was the largest subgroup, which included 39% (*n* = 6084) of the female employees. For this profile, the seven job quality index scores were generally shown to be average.

The “footloose” profile was a subgroup to which 9% (*n* = 1335) of the female employees belonged. Unlike the high-flying profile, low work intensity and high quality of working time were noticeable in this group; however, these employees generally had the lowest income and worst job prospects out of all the subgroups.

The “strict” profile, to which 18% (*n* = 2855) of the female employees belonged, showed the lowest quality of working time compared to other profiles. These female employees’ physical environment and work intensity (reversed) scores were lower than average; however, their earning, prospects, skills and discretion, and social environment scores were slightly higher than average.

The “manual” profile was the smallest group, comprising 4% (*n* = 636) of the female employees. The employees in this group showed below average scores for most indicators. In particular, physical environment scores were the lowest and work intensity was the strongest, compared to the other profiles. Job prospects were also shown to be somewhat poor.

### 3.3. Female Employee Distribution in Job Quality Profiles

**Figure 3:**
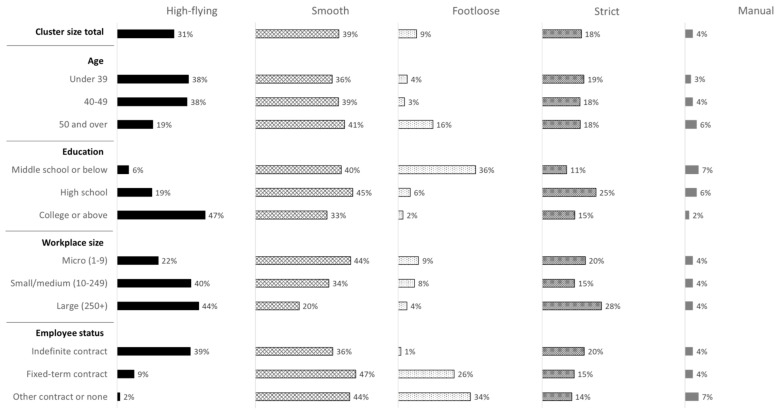
Job quality profiles by demographic characteristics (% of employees in each category).

Compared with other age groups, female employees aged 49 years or younger showed a relatively large distribution in the “high-flying” subgroup, while those aged 50 years or older showed a larger distribution in the “footloose” subgroup. Regarding education level, female employees who were middle school graduates or below were very strongly represented in the “footloose” profile (36%), while those with college degrees or above most frequently belonged to the “high-flying” profile (47%). High school graduates showed a relatively large distribution in the “strict” profile. In relation to workplace size, most employees at micro-companies had “smooth” jobs, while the majority of employees at large companies had “high-flying” or “strict” jobs. By employment status, regular employees were relatively more frequently distributed in the “high-flying” profile, and temporary or other employees showed relatively large distributions in the “footloose” profile (Figure 3).

**Figure 4:**
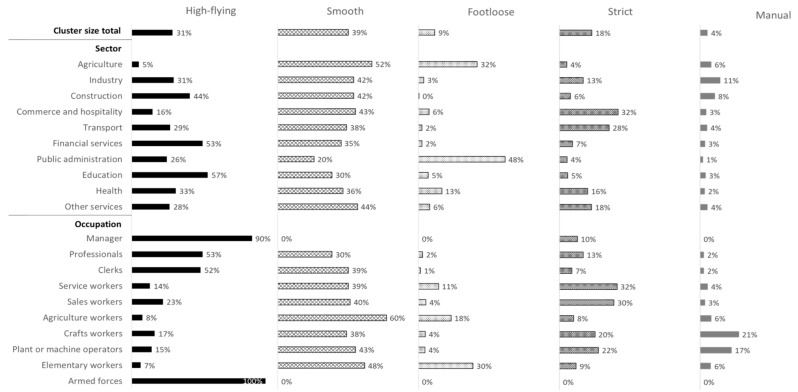
Job quality profiles by sector and occupation (% of employees in each category).

By sector, employees in the agriculture and public administration fields showed relatively large distributions in the “footloose” profile, and about half of the employees working in financial services or education belonged to the “high-flying” profile. Those with industry or construction jobs showed relatively large distributions in the “manual” profile. By occupation, most managers had “high-flying” jobs, as did approximately half of the professionals and clerks. Service or sales workers were highly represented in the “strict” profile. Additionally, workers in crafts and related trades and plant or machine operators were typically represented in the “manual” profile. Elementary workers showed a large distribution within “footloose” jobs (Figure 4).

### 3.4. Association between Job Quality Profiles and Work-Life Balance

**Figure 5:**
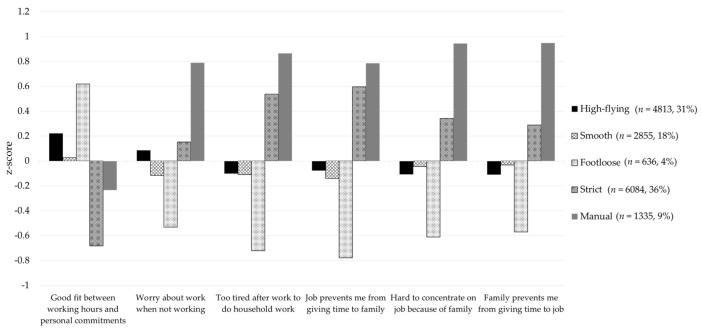
Associations between job quality profiles and work-life balance.

All female employees showed statistically significant differences in work-life balance by job quality profile (*p* < 0.01; Figure 5). Employees with the “footloose” profile showed not only high work-life balance between working hours and personal time but also the most positive work-life balance, as they had less work-family conflict. However, employees with the “manual” profile showed the highest level of conflict between work and family. The “strict” profile showed the worst fit between working time and personal time and a high level of work-family conflict. Employees with either “high-flying” or “smooth” profiles showed average levels for most indicators.

## 4. Discussion

This study started from the limitations of previous studies, which did not reflect the multifaceted aspect of the quality of work-life balance of South Korean female employees. Using the KWCS, which is a representative survey of Korean workers, we classified latent profiles to include various variables indicating job quality and analyzed the distribution of demographic and occupational characteristics and the difference in work–life balance among the profiles. The results suggest an alternative to the research flow, which has so far focused only on specific working conditions. In addition, they illustrate the need to attend to job quality as experienced by female employees and, in particular, policies and practical interventions reflecting the characteristics of each profile with regard to the work–life balance of female employees.

Female employees were classified into five profiles—"high-flying”, “smooth" “footloose”, “strict”, and “manual”. Although direct comparisons are difficult due to cultural differences and the targeting of only female employees, these results are slightly different from those in the sixth EWCS, where the profiles were classified as “high-flying”, “smooth running”, “active manual”, “under pressure”, and “poor quality.” The “under pressure” profile in the EWCS showed a low social environment; however, South Korean female employees showed similar social environment levels across all five profiles. Furthermore, the “poor quality” profile in the EWCS was low for all seven job quality indices; however, it did not appear among the profiles in the present study. However, South Korean female employees were categorized into “footloose” and “strict” profiles, which had the highest and lowest working time quality, respectively.

The employees in the “high-flying” profile simultaneously experienced a high level of job demand and resources. They accounted for about one-third of all female employees. Many of these included highly educated employees, managers, and professionals. These employees worried about work when they were at home because their jobs required a high level of skill; however, their work-life balance should have been promoted since they had many job resources such as work autonomy, social support, and high wages [29,49]. However, research results are contradictory on this topic, indicating that the higher the income and position, the more conflicts there are between work and life, may be due to the tendency to be more devoted to work [50,51]. Thus, the provision and utilization of many job resources that can alleviate the foregoing stress should be promoted. Furthermore, although “women’s” entry into managerial and professional positions is gradually expanding as the level of education increases, the proportion of female managers in South Korea is still low at 21% [52]. In the case of workplaces with a high proportion of female managers, support for work-life balance appears to be well equipped [52]. Therefore, a support strategy is required to promote the advancement of female workers to management positions.

The “smooth” profile formed the largest subgroup, with 36% of employees. This group has moderate job demands and resources. The participants in this group were mainly over 50 years of age and engaged in small workplaces or in the agriculture or elementary field. Although this profile generally showed average scores across all seven job quality indices and work–life balance, as the employment activities of middle-aged and older workers are becoming more important due to aging and the low birth rate, efforts to improve the quality of their work are required. In South Korea, the retirement age of 60 is mandatory. The quality of employment and wage levels are greatly reduced as middle-aged and older workers leave their main jobs without preparing for retirement, and many are re-employed in temporary, daily, and non-regular jobs [53]. In this environment, job satisfaction and the level of wages in preparation for later years will enable middle-aged and older workers to continue working, and this is also linked to work–life balance [54,55,56]. Therefore, the design and arrangement of suitable jobs, the provision of education and training, and continuous support at the government level are necessary to enhance the job satisfaction of middle-aged and older workers.

The “footloose” profile showed the most positive work-life balance. The employees in this profile had the lowest earnings and prospects and the highest working time quality among the job resources. However, among the job demands, skill and discretion were the lowest and work intensity (reversed) was the highest. The work-life balance of the footloose profile group was the highest among all profiles, and the work-family conflict was the lowest. This is consistent with the JD-R model theory that the level of work–life balance of workers is not simply the absolute value of job demand or job resources; instead, a moderate effect between the two is more important [34]. This profile had the lowest earnings and prospects, suggesting that a lower contribution to overall household income was associated with better well-being [57]. Therefore, some female employees may do things using their careers, or earn little money, but do not have many financial difficulties. However, most of the employees in this profile were temporary or daily employees and worked jobs requiring low-skilled labor. Additionally, there were many employees who received low wages. This is likely connected to the situation of female employees in South Korean society [21]; thus, the good balance between work and life in this study may not be reflected in reality. Furthermore, the job insecurity can lead to anxiety in workers and negatively affect work-family balance [58]. Therefore, it is necessary to conduct more empirical studies on the “footloose” profile.

The “manual” profile showed the lowest scores for physical environment and work intensity and the highest level of work-family conflict. A poor physical workplace environment negatively affects employees’ health, which can increase work-family conflict by increasing employees’ physical and psychological stress [24,59,60,61]. Furthermore, given that mainly craft employees and plant or machine operators belonged to this profile, the intensity of work that must be performed in accordance with production goals could also induce job stress, negatively affecting work-life balance [62]. Therefore, to promote work-life balance, it is necessary to provide systematic management and education for employees’ health and create an appropriate workplace environment.

The “strict” profile had good job resources (prospects, earnings); however, the level of job demand (the lowest quality working time) was also high, leading to a poor fit between working hours and personal commitments. Moreover, this could negatively affect employees’ families due to the physical and mental stress caused by long working hours [29,63,64]. Shift working time may also have affected such outcomes. The possibility of adjusting working time in the commerce and hospitality fields, to which the employees with this profile mainly belonged, was found to be very low-approximately 20%—indicating that working time and location were relatively inflexible in these types of employment [65]. When the nature of the job makes it is difficult to introduce flexible work arrangements, it is necessary to consider a plan to give employees the right to choose vacation dates first, and allow them to use an alternative vacation when their days off and holidays overlap.

## 5. Conclusions

We classified latent profiles based on job quality indices according to the JD-R model and investigated differences in demographic and occupational characteristics and work–life balance between profiles among female employees in South Korea. Although the latent profiles in this study, analyzed using data from the fifth KWCS, cannot be generalized and applied to all workers, the results of this study enabled the identification of the working conditions and sociodemographic and occupational characteristics reflected by each profile as well as which characteristics of organizations should be improved for balance. As mentioned in the discussion, ensuring job security, preparation of a work-related mental and physical stress management system, support for long-term vacations, reflection of preferences for work schedules, provision of social support systems, and provision of educational opportunities for continuous self-development required according to the characteristics of each profile can be considered as strategies for the work–life balance of South Korean female workers. In addition, for these interventions to be carried out in harmony with the strategies for promoting work–life balance currently being implemented by companies and the government, policy and social awareness should be spread and supported.

This study has the following limitations. First, only the variables included in the raw data were used in the secondary data analysis. Although working conditions were divided into seven job quality indices, and efforts were made to approach them as comprehensively as possible, it was difficult to determine latent groups that were not included in the raw data. Additionally, although the occupational characteristics of the classified profiles were identified, family characteristics, such as marital status, number of children, and how respondents utilized their leisure time, could not be identified. Therefore, studies to analyze potential groups using other data sources should be conducted in the future. Second, although various policies are being implemented to promote work-life balance in South Korea, this study could not measure whether the policies had been applied for the participants. To prepare more effective policies for work-life balance, additional studies will be required to consider whether current policies are applied as well as their effects. Finally, as this was a cross-sectional study, the associations between latent groups and work-life balance were identified; however, causal relationships could not be determined. Therefore, to develop and apply strategies to promote work-life balance according to each latent profile, longitudinal studies to identify causal relationships should be conducted.

## Figures and Tables

**Table 1:** Model fit statistics of latent profile models (*N =* 15,723).

No. of Profiles	BIC	Entropy	LMR-LRT	Latent Profile Proportions (%)					
				1	2	3	4	5	6
2	768,383.82	0.882	<0.001	12.3	87.7				
3	764,042.05	0.810	<0.001	12.8	65.5	21.7			
4	761,201.32	0.834	<0.001	4.9	12.3	19.8	62.9		
5	758,586.04	0.745	<0.001	18.2	4.0	8.5	38.7	30.6	
6	757,048.45	0.777	<0.001	3.7	8.3	17.9	34.8	34.8	0.5

Abbreviation: BIC, Bayesian information criterion; LMP-LRT, Lo-Mendell-Rubin adjusted likelihood ratio test.

## Data Availability

Publicly available datasets were analyzed in this study. This data can be found here: https://oshri.kosha.or.kr/oshri/researchField/downWorkingEnvironmentSurvey.do (accessed on 26 April 2019).
